# ANN-Based Soft Sensor to Predict Effluent Violations in Wastewater Treatment Plants

**DOI:** 10.3390/s19061280

**Published:** 2019-03-13

**Authors:** Ivan Pisa, Ignacio Santín, Jose Lopez Vicario, Antoni Morell, Ramon Vilanova

**Affiliations:** Department of Telecommunications and Systems Engineering, Escola d’Enginyeria, Universitat Autònoma de Barcelona, 08193 Bellaterra, Spain; ignacio.santin@uab.cat (I.S.); jose.vicario@uab.cat (J.L.V.); antoni.morell@uab.cat (A.M.); ramon.vilanova@uab.cat (R.V.)

**Keywords:** wastewater treatment plants, artificial neural networks, long-short term memory cells, soft sensors

## Abstract

Wastewater treatment plants (WWTPs) form an industry whose main goal is to reduce water’s pollutant products, which are harmful to the environment at high concentrations. In addition, regulations are applied by administrations to limit pollutant concentrations in effluent. In this context, control strategies have been adopted by WWTPs to avoid violating these limits; however, some violations still occur. For that reason, this work proposes the deployment of an artificial neural network (ANN)-based soft sensor in which a Long-Short Term Memory (LSTM) network is used to generate predictions of nitrogen-derived components, specifically ammonium ($S_{NH}$) and total nitrogen ($S_{Ntot}$). $S_{Ntot}$ is a limiting nutrient and can therefore cause eutrophication, while nitrogen in the $S_{NH}$ form is toxic to aquatic life. These parameters are used by control strategies to allow actions to be taken in advance and only when violations are predicted. Since predictions complement control strategies, the evaluation of the ANN-based soft sensor was carried out using the Benchmark Simulation Model N.2. (BSM2) and three different control strategies (from low to high control complexity). Results show that our proposed method is able to predict nitrogen-derived products with good accuracy: the probability of detecting violations of BSM2’s limits is 86–94%. Moreover, the prediction accuracy can be improved by calibrating the soft sensor; for example, perfect prediction of all future violations can be achieved at the expense of increasing the false positive rate.

## 1. Introduction

Wastewater treatment plants (WWTPs) form an industry devoted to processing and reducing the pollution present in urban residual water. Their goal is to reduce the incoming water’s pollutant concentrations and, therefore, to preserve the natural resources and the environments into which treated water is discharged. Concentrations of pollutants, which consist of components derived from nitrogen and phosphorus, are reduced by means of highly complex biological and biochemical processes. In addition, the maximum allowed pollutant concentrations present in a WWTP’s effluent are regulated by certain limits established by local administrations.

The main aim of imposing limits is to assure that incoming water is being treated, and therefore, the pollutant concentrations are being reduced. By doing so, the environment into which effluent is dumped can be preserved, and its progressive destruction due to the spilling of contaminated waters into watercourses can be prevented. As a result, WWTPs are punished when violating these limits. Those punishments depend on where WWTPs are located, and each administration is responsible for adapting them to their necessities. For instance, European regulations (European Directive 91/271 [1]) define different thresholds for the different WWTP effluent’s components. Moreover, these limits are even more restrictive if WWTPs are placed in sensitive areas. Thus, limits are established not only to determine whether a WWTP has to be punished because it is not treating the incoming water but also to ensure that the environment into which treated waters are dumped is preserved.

The International Water Association (IWA) has developed highly complex and nonlinear mathematical models able to replicate a WWTP’s behavior. The most well known and established one is the Activated Sludge Model N.1 (ASM1) [2], which models the biochemical and biological processes performed in WWTPs related to nitrogen compound dynamics. It has also been adopted in the design of certain simulation scenarios, such as the Benchmark Simulation N.1 (BSM1) and Benchmark Simulation N.2 (BSM2). BSM1 emulates the behavior of a generic urban WWTP’s water line [3], but it does not replicate the sludge treatment performed outside of this line. To solve this, BSM1 has been enhanced by the appearance of BSM2, which includes BSM1 as well as the sludge treatment process performed outside of the water line [4]. Both simulation scenarios seek the elimination of carbon and nitrogen components from the incoming water. In terms of the regulations applied in these frameworks, BSM1 and BSM2 implement their own effluent limit concentrations [5] since they are frameworks offering generality, easy comparison, and replicable results.

Artificial neural networks (ANNs) [6] have arisen as data-driven methods able to generate mathematical models of high-complexity processes such as the ones carried out at WWTPs. For instance, ANNs were applied in [7] to generate a mathematical model of ASM1’s behavior in BSM1 and BSM2 scenarios. Only the WWTP’s influent and effluent data are required. In addition, BSM1 and BSM2 are adopted to test certain control strategies in order to maintain the pollutant levels under their limits. For instance, in [8], model predictive control (MPC) and fuzzy logic control strategies were tested together in a BSM2 scenario. Consequently, the effects of a control strategy should be taken into account when approaching the modeling of ANNs since they are observable in the WWTP’s data effluent.

In some works, ANNs were adopted as soft sensors to predict certain parameters by combining available measurements [9]. Soft sensors only require available measurements in order to perform predictions of unmeasured process values and/or parameters. Consequently, they have arisen as a low-cost alternative to expensive hardware. This fact motivates its adoption in such different industries as refineries and WWTPs. In [9,10], ANN-based soft sensors were deployed to obtain the offline measurement of certain processes (measurements that cannot be obtained directly from the plant and require certain laboratory analyses) from online ones (real-time available measurements) in a refinery’s distillation column. The same objective was sought in [11], where an ANN-based soft sensor was deployed in a WWTP plant to predict offline and hard-to-measure values from available ones.

On the other hand, the increasing interest in the Internet of Things (IoT) and Industry 4.0 [12] has also motivated the adoption of ANNs for different purposes in WWTP systems. For instance, there are many works in which ANNs were considered to monitor or predict some WWTPs’ parameters. In [13], the chemical oxygen demand (COD), suspended solids (SS), and the aeration tank’s dissolved oxygen concentrations ($S_{O}$) were predicted and tracked by means of three different multiple layer perceptron (MLP) neural networks. Different nets’ configurations were considered, where the best one adopted 20 neurons in a unique hidden layer. It produced a mean absolute percentage error (MAPE) in the prediction of around 4.48%. Another example is the one observed in [14], where the authors proposed an MLP structure to predict the COD, total suspended solids (TSS), and the biochemical oxygen demand ($BOD_{5}$) considering past values of the same parameters. The correlation coefficient (*R*), which consists of the square root of the determination coefficient ($R^{2}$), was adopted as a performance metric. Results showed an *R* of around 0.93, which means that the MLP’s predictions were quite correlated to real values.

To study the application of ANNs to predict effluent concentration at WWTPs, Foscoliano et al. adopted a recursive neural network (RNN) that forecasted the WWTP’s nutrient concentrations and then fed an MPC-based control strategy to maintain the pollutant concentrations under the maximum levels [15]. Results were obtained by means of BSM1 model simulations. Another approach was shown by Manu et al., who adopted a neural network to predict the performance of a WWTP plant in removing total Kjeldahl nitrogen (TKN), as proposed in [16]. The predictions then fed a fuzzy logic strategy. Results showed that a correlation coefficient of around 0.97 was achievable. In [17], two MLPs were adopted to predict the ammonia ($S_{NH,e}$) and total nitrogen ($S_{Ntot,e}$) concentrations in the effluent and determine whenever a violation of their limits was likely to occur. When detecting a violation, an (MPC + fuzzy logic)-based control strategy was activated automatically. In that manner, a reduction of 63.41% of $S_{Ntot,e}$’s violation time was achieved with respect to [18]’s control strategy. However, the MLPs’ predictions in [17] correspond to the effluent’s maximum value observed within a day. Even though predictions were performed online (in real time), the MLP was trained using offline data: the maximum effluent’s value was considered to be MLP’s output data. In that sense, the time correlation between influent and effluent was broken because predictions do not tell when a peak of pollution will be exactly produced. Thus, the control strategy should be applied throughout the day instead of a few moments before the peak is really observed. The common point among these works is the fact that control strategies have been adopted to ensure that effluent concentrations are upheld below the limits; however, some violations still occur. For instance, Jeppsson et al. showed violations of $S_{NH,e}$ equal to 0.41% of the WWTP’s operational time (1 year) and equal to 1.18% in terms of $S_{Ntot,e}$ [18]. Those percentages were translated into violations of ammonium lasting 1.5 days and violations of total nitrogen for around 4.3 days.

The literature shows that ANNs have been widely used to predict certain WWTP measurements. Some studies have adopted MLP networks, which do not preserve the measurement’s time correlation, whereas others have adopted RNNs to preserve it. However, offline measurements are considered to be an RNN’s input data. Therefore, predictions in real time cannot be obtained since offline measurements are not available without performing laboratory analyses. For that reason, we propose the design and implementation of an Effluent Concentration and Alarm Prediction System (ECAPS) that is based on an ANN-based soft sensor. This soft sensor adopts Long-Short Term Memory (LSTM) cells, a type of RNN. They predict the concentration of the effluent’s nutrients, specifically ammonium $S_{NH,e}$ and total nitrogen $S_{Ntot,e}$, two of the most difficult nitrogen-derived concentrations to reduce. Predictions, which are performed in real time adopting BSM2 online available data, determine whenever an effluent limit is prone to violation. In addition, the predictions feed existing control strategies to let them actuate in advance [17]. In this fashion, possible violations of effluent limits can be detected and minimized to better preserve the environment. Furthermore, this yields a reduction in the WWTP’s overall cost since the cost of deploying expensive hardware to measure offline effluent nutrients ($S_{Ntot,e}$) [11] is also reduced.

In summary, the main contributions of this work are

- The design of a soft sensor based on ANNs (LSTM structures) to predict WWTP’s effluent concentrations.
- The treatment of online data as the unique source of information to predict effluent limits in real time.
- The application of data preprocessing techniques to improve the LSTM predictions.
- The establishment of a prediction system able to attain a maximum MAPE of 22.65% and provide a minimum ammonium violation detection probability of around 89.02%.

## 2. Materials and Methods

### 2.1. Benchmark Simulation N.2

The proposed system is based on the application of ANN-based soft sensors to predict the effluent values given certain WWTP’s input measurements. In that sense, influent and effluent measurements are required to train the neural networks. They are generated through the usage of the BSM2 simulation scenario. It has its own simulation protocol that takes into account a defined influent profile. This profile corresponds to the influent parameters observed during a period of 609 days. These are sampled with a rate of one sample every 15 min. In addition, dry, rainy and stormy weather are taken into account in the influent profile. In terms of the simulation procedure, BSM2 requires a calibration process, which is performed by means of an initial 200-day dry-weather influent profile. Once the model is calibrated, the 609-day influent profile can be simulated.

#### 2.1.1. Layout

BSM2 not only considers the water line in the BSM1 scenario but also the sludge treatment process. Consequently, two clearly differentiated sections can be observed. The first one is devoted to reducing the pollutant concentrations of water by means of biochemical and biological processes, and the second one aims to treat the WWTP’s sludge.

In that sense, the water’s biological treatment is produced by biochemical and biological processes in the activated sludge reactors, which consist of a set of five biological reactor tanks: two anoxic and three aerobic. The behavior of the biochemical and biological processes is defined by the ASM1 model [2]. Among all the defined processes, we focus on nitrogen reduction, which is based on nitrification and denitrification. Nitrification consists of the oxidation of ammonium ions into nitrate, which is processed in the denitrification process. Denitrification transforms the nitrate into nitrogen and other gaseous products [19].

**Figure 1:**
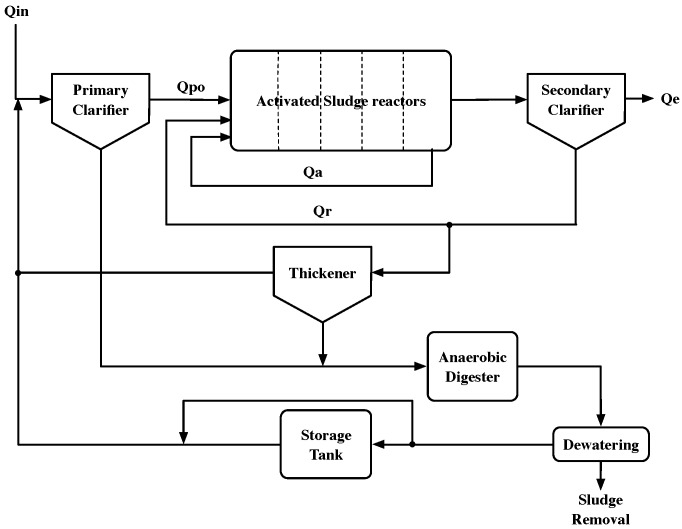
Benchmark Simulation Model N.2 (BSM2) model for a biological wastewater treatment plant (WWTP).

The sludge treatment process is performed by different modules (see Figure 1): the primary and secondary clarifiers, the anaerobic digester, the dewatering module, and the storage tank. The primary and secondary clarifiers are devoted to performing the sedimentation process following the layered structure proposed in the Takácks model [20]. The sludge obtained in the clarifiers is treated in the anaerobic digester and either removed in the dewatering module or saved in the storage tank. Finally, when it is required, sludge can be refilled in the activated sludge reactors from the storage tank.

In terms of the flow rates, the BSM2 scenario is designed to account for an average flow rate of 20,648.36 m${}^{3}$/day, a volume of 1500 m${}^{3}$ for each anoxic tank, and a volume of 300 m${}^{2}$ for each aerated one. Consequently, the average retention time for WWTP plants following the BSM2 scenario is set to 14 h. This is considered in the process of predicting the effluent concentration because it determines the interval for which the prediction is generated. Finally, the main flows considered in the scenario are the following ones: $Q_{in}$ is the influent rate, $Q_{po}$ is the primary clarifier’s flow rate, $Q_{a}$ corresponds to the internal recirculation flow rate (understood as the quantity of aerated flow going from the fifth tank to the first one), Qr is defined as the sludge’s internal recycle flow rate, which moves part of the sludge from the second clarifier to the first anoxic tank, and finally, $Q_{e}$ corresponds to the WWTP’s effluent.

#### 2.1.2. BSM2’s Effluent Pollutant Limits

The BSM2 simulation scenario, on which this work is based, not only describes and defines its own architecture and behavior but also implements its own limit regulations. Since one of BSM2’s objectives is to offer a framework that allows for generality, easy comparison, and replication of results, BSM2’s effluent pollutant limits do not follow any special regulation or local legislation [5].

BSM2 concentrations should always be maintained below the levels shown in Table 1, which shows the limits of the pollutants present in discharged water. As it is observed, they correspond to biochemical oxygen demand ($BOD_{5}$), chemical oxygen demand (COD), total suspended solids (TSS), ammonium concentration ($S_{NH,e}$), and total nitrogen concentration ($S_{Ntot,e}$). Therefore, a violation occurs whenever a concentration exceeds the established limits and results in the application of sanctions to the WWTP [21]. In this context, the concentrations of interest correspond to ammonium ($S_{NH,e}$) and total nitrogen ($S_{Ntot,e}$), which are two of the most difficult concentrations to manage; carbon and suspended solid components are addressed by the usual control and operational strategies [17]. Moreover, $S_{Ntot.e}$ is a limiting nutrient and can therefore cause eutrophication, while nitrogen in the $S_{NH,e}$ form (ammonium) is toxic to aquatic life [21,22].

It is worth noting that the concentrations of pollutants derived from phosphorus are not considered by BSM2 regulations. This is motivated by the fact that BSM2 reactor models are based on ASM1 [2], which does not take into account phosphorus components. Instead, Activated Sludge Models 2 and 2d (ASM2 and ASM2d) would be required if phosphorus were to be taken into account [23].

#### 2.1.3. Control Strategies

**Figure 2:**
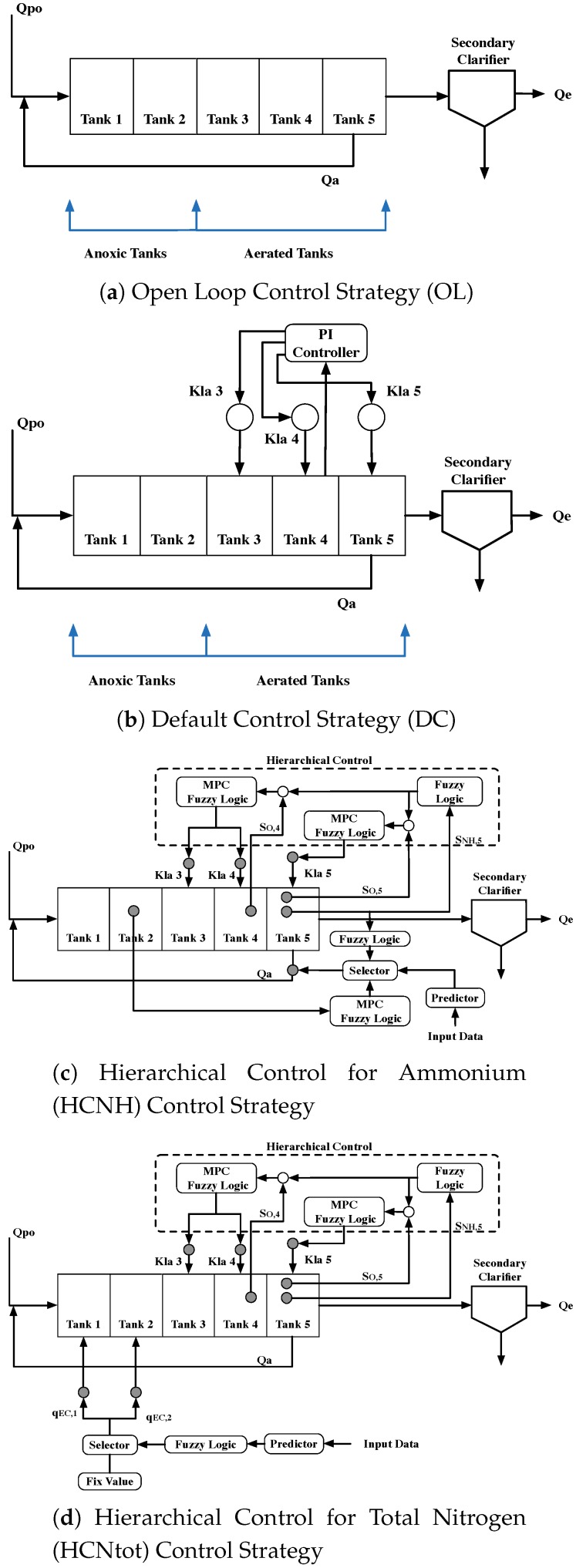
Control strategies. Open Loop is the one considering the lowest control level, whereas the highest one is considered in the HCNH and HCNtot Control Strategies.

In order to test the behavior of the proposed ANN-based soft sensor system, three different scenarios of control are considered: (i) Open Loop (OL), (ii) Default Control (DC), and (iii) Hierarchical Control (HC). The OL control strategy (see Figure 2a) corresponds to the lowest level of control since no control scheme or strategy is adopted.

BSM2’s default control strategy (see Figure 2b) is in charge of keeping the dissolved oxygen in each aerated tank ($S_{O,x}$—*x* denotes the respective aerated tank) at the desired level. This is performed by adopting a proportional integral (PI) controller, which manages the oxygen transfer coefficient ($K_{l}a$ 3, $K_{l}a$ 4, $K_{l}a$ 5) associated with each one of the WWTP’s aerated tanks (third, fourth, and fifth tanks) [18,24].

Finally, the highest considered control level corresponds to a hierarchical-based approach (see [17] for further details). It has two differentiated parts. The former is focused on the control of $S_{O,x}$ concentrations (as in the default control strategy), but it adopts a hierarchical control strategy based on MPC and fuzzy logic controllers and considers the ammonium concentration in the last tank ($S_{NH,5}$). The latter corresponds to an additional predictive control that is activated when effluent violations are predicted. Two different structures are defined, depending on the violation to control: the Hierarchical Control for Ammonium (HCNH) (see Figure 2c) and the Hierarchical Control for Total Nitrogen (HCNtot) (see Figure 2d). The first one modifies the internal recirculation flow ($Q_{a}$), whereas the second modifies the value of the external carbon added to tanks 1 and 2 ($q_{EC,1}$ and $q_{EC,2}$).

### 2.2. ANN-Based Soft Sensor

The main goal of a soft sensor is to form predictions of unmeasured variables by using mathematical algorithms and online available data. Therefore, they have arisen as a solution to the deployment of expensive hardware sensors. They also allow the obtention of unmeasurable variables and offline measurements as if the data were online [9]. In this work, the soft sensor approach is considered to predict violations of effluent concentrations. If it is not considered, prediction becomes a difficult task. In particular, the proposed soft sensor in this work is based on the implementation of an ANN to combine the available data. The advantage of ANN-based soft sensors is the ability to perform complex nonlinear operations. Besides this, these mechanisms are easily tuned. Since they are based on ANNs, the parameters of the system are obtained by means of a training procedure based on input and output data from the process to sensor.

**Figure 3:**
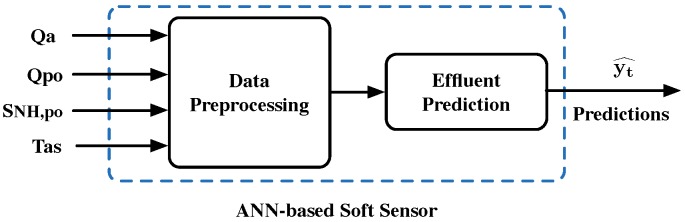
Artificial neural network (ANN)-based soft sensor. Predictions ($\hat{y_{t}}$) can be either ammonium ($S_{NH,e}$) or total nitrogen ($S_{Ntot,e}$) concentrations.

In this work, the ANN-based soft sensor is responsible for predicting nitrogen-derived components: ammonium ($S_{NH,e}$) and total nitrogen ($S_{Ntot,e}$). To do so, the ANN-based soft sensor considers the implementation of ANNs and specifically LSTM cells. It also includes the Data Preprocessing block, where data are preprocessed and prepared before being fed into the soft sensor’s Effluent Prediction block (see Figure 3). Thus, the ANN-based soft sensor can be observed as a black box in which rough data enters and predictions of the desired effluents’ concentrations are returned. The point here is that soft sensors’ input and output data consist of online measurements with the exception of total nitrogen ($S_{Ntot,e}$). It cannot be directly obtained since it corresponds to an offline measurement. So, thanks to the soft sensor, measurements of $S_{Ntot,e}$ can be given in real time.

The ANN-based soft sensor’s input and output data were generated by means of the BSM2 simulation scenario. Even though a large number of measurements can be considered, the available influent, internal, environmental, and effluent measurements shown in Table 2 were selected. Input data correspond to the influent, internal, and environmental measurements, whereas output data correspond to the effluent pollutant concentrations, specifically $S_{NH,e}$ and $S_{Ntot,e}$, and they are sampled every 15 min. These measures are considered because of their roles in the nitrification and denitrification processes and therefore in the effluent pollutant concentration.

### 2.3. Data Preprocessing

Before being used in the ANN-based soft sensor’s training process, the WWTP’s available data have to be preprocessed. Therefore, an exploratory analysis of available data was performed to observe the type of measurements we are addressing. Their range, mean, and variance were computed in order to obtain an initial perspective of the data topology. For instance, some of the WWTP’s measurements are widely heterogeneous: $S_{Ntot,e}$’s maximum value is below 25 when HC strategies are applied, whereas $Q_{po}$’s minimum value is over 5500. Consequently, the feature standardization normalization technique was applied in order to decrease the heterogeneity in the data [25]: the distribution mean and standard deviation were computed for each input parameter, and therefore, data were normalized to zero-mean and unit-variance by adopting Equation (1).

(1) $$x_{norm}=\frac{x-\bar{x}}{\sigma_{x}}$$

where $\bar{x}$ is the mean and $\sigma_{x}$ is the standard deviation.

**Figure 4:**
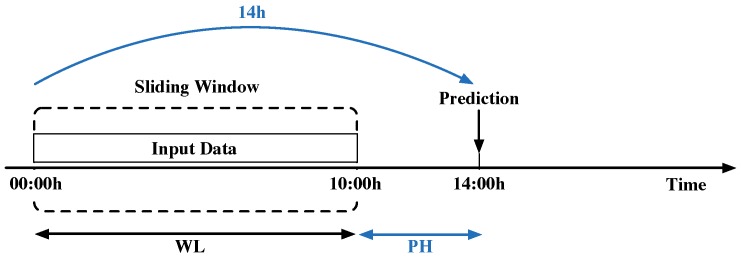
Sliding window structure. Notice that window length (WL) and prediction horizon (PH) correspond to 10 and 4 h.

On the other hand, the considered data correspond to time-dependent measurements. These measurements feed the ANN-based soft sensor to predict the WWTP’s effluent pollutant concentrations. Consequently, measurements have to be organized. In order to perform this, we use 10 h of input data (window length equals 40 samples—each hour corresponds to 4 samples) to predict effluent values with a prediction horizon (PH) of 4 h (see Figure 4).

**Figure 5:**
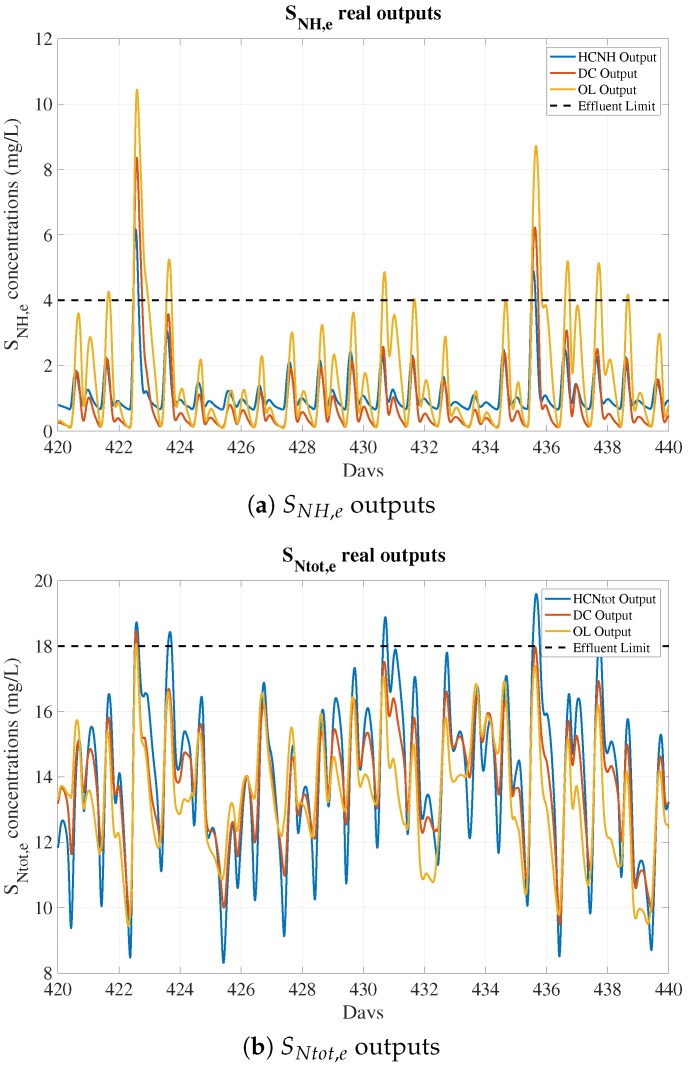
Real outputs for HC, DC, and OL control strategies. Notice that nearly all days are below the effluent limit, showing that measurements above the limits are clearly underrepresented.

Furthermore, the purpose of the proposed soft sensor is to perform predictions of effluent values and, more importantly, determine whenever a violation of the effluent limits takes place. In that sense, having balanced data means that approximately half of the effluent values are over the effluent limit and half of them are below. However, data are not always balanced, as is shown in Figure 5. Only 0.23% of $S_{NH,e}$ and 2.56% of $S_{Ntot,e}$ measurements are above the effluent limits. Therefore, from the effluent limit violation perspective, this is a situation clearly underrepresented. Cross-validation helps in this situation as long as different training runs of the ANN’s model are performed using different splits of data. Then, the training that offers the best performance in terms of predictions determines the model parameters to adopt. Notice that model parameters correspond to configuration variables that are internal to the model and tuned through the model training process.

The data preprocessing explained here corresponds to the one applied when dealing with the extreme case of a one-sample averaging time-window policy. Thus, prediction of any effluent violation can be performed whenever a new sample is gathered. However, the proposed soft sensor can be re-defined in order to fulfill other operational points or policies. It is just a matter of selecting the properly averaging time window, which is a decision to be made by plant operators. For instance, if the European Directive 91/271 has to be fulfilled, the soft sensor only has to change its averaging time window. It should take into account 24 h average concentrations [1] to determine whether the maximum number of allowed failing samples is exceeded.

### 2.4. LSTM-Based ANN

ANNs were adopted as the main method to generate soft sensor predictions (WWTP’s effluent predictions) because they are able to model nonlinear models such as ASM1. ANN structures consist of a set of layers, where each one presents a number of hidden neurons that are characterized by the activation function they adopt. Sigmoid, hyperbolic tangent, and linear activation functions are three of the most used activation functions ([26], (Section 1.3)).

In addition, different architectures of ANNs are defined depending on how their units or neurons interconnect, for instance, feed-forward neural networks (FFNNs) ([26], (Chapter 5)) and recurrent neural networks (RNNs) ([6], (Chapter 10)), among others. On one hand, FFNNs are ANNs wherein connections between layers and neurons are always in the same direction ([26], (Chapter 5)) and [27]. RNNs are ANNs wherein connections between hidden neurons form a directed cycle. Thus, information about past events can be intrinsically used for future predictions. These have been successfully applied to model continuous signals or nonlinear systems ([6], (Chapter 12)).

The connections in an ANN are adopted or trained so that the network matches known input–output pairs (supervised learning) in the best possible way, i.e., with minimum error. The strength of ANNs’ parameter training process is that its basis is an iterative process that depends on the error made. This process is performed using the well-known back-propagation (BP) algorithm, which is responsible for updating the ANN’s parameters toward the reverse of the cost function’s gradient ([6], (Chapter 6)). In RNNs, a BP variant, called back-propagation through time (BPTT) is available. However, BPTT presents a drawback when it is applied to RNNs: the exploding or vanishing gradients problem [28]. LSTM cells are alternative structures, called gated networks, that overcome this problem ([6], (Section 10.10)).

Finally, ANNs adopt certain regularization techniques to avoid overfitting. Overfitting occurs when the neural network complexity is so high that it is able to memorize the right output for each input without really learning a model. In such a case, the performance of the ANN significantly decreases with new data. To solve this problem, the L2 penalty regularization technique is used. L2 is based on the addition of extra penalties to the ANN’s parameters, thus reducing the network capacity to perfectly match training examples ([6], (Section 7.1.1)).

**Figure 6:**
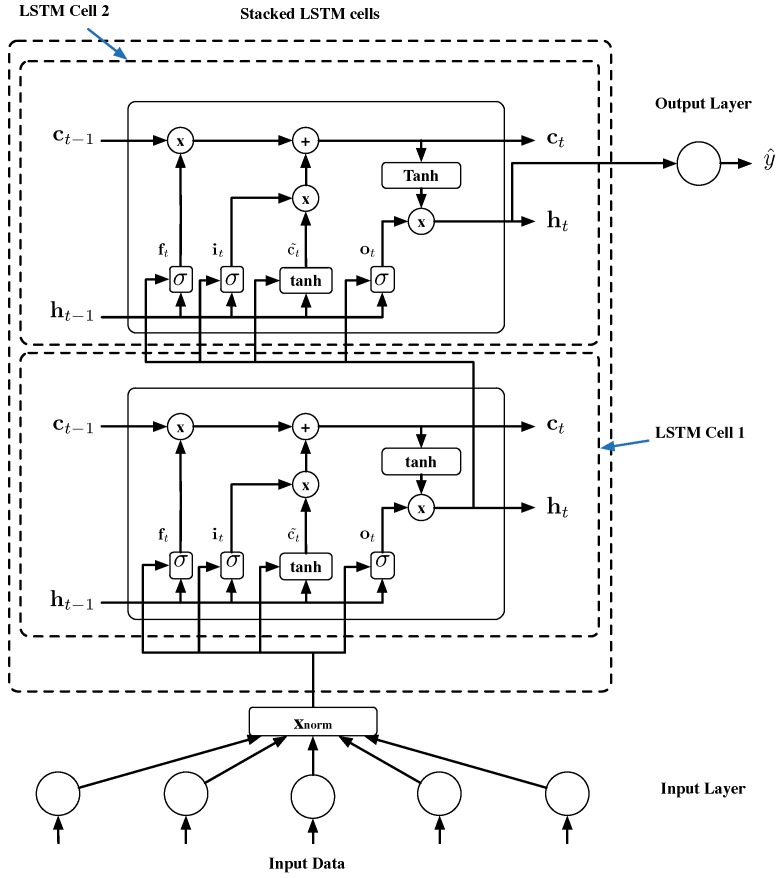
Two stacked Long-Short Term Memory (LSTM) cells. ${\hat{y}}_{t}$ corresponds to the prediction which can be either $S_{NH,e}$ or $S_{Ntot,e}$ concentrations.

In this work, among the different ANN strategies, LSTM cells were used to model the temporal behavior and dependence between WWTP’s inputs and outputs. This is because of their capacity in modeling time-series and time-dependent values ([6], (Section 10.10)) and [29]. Their structure is observable in Figure 6, which shows two-stacked LSTM cells.

Each LSTM cell has three inputs and two outputs, where the inputs correspond to

- $x_{t}$: the input data vector ($X_{\mathrm{norm}}$ in the case of this work).
- $h_{t-1}$: the previous output of the cell.
- $c_{t-1}$: the state of the cell (the memory). It stores information about what the cell has seen previously.

The outputs correspond to

- $h_{t}$: the current output of the cell. When cells are stacked, the output of the cell below is the input of the cell above. In Figure 6, $h_{t}$ of LSTM Cell 1 equals $x_{t}$ of LSTM Cell 2.
- $c_{t}$: the updated memory of the cell.

From an operational point of view, LSTM cells are characterized by having different ANNs that are in charge of the memory and data management ([6], (Section 10.10)). These are

- Input Gate ($i_{t}$): sigmoid layer that takes into account previous output and current input of the cell to decide on the modification of the inner cell state. It is described mathematically as follows:
  (2) $$i_{t}=\sigma \left(W_{i}^{T}x_{t}+U_{i}^{T}h_{t-1}+b_{i}\right)$$
- State candidates ($\tilde{c_{t}}$): tanh layer that takes into account the previous output and current input to determine the new candidates of the cell state. New candidates are computed as:
  (3) $$\tilde{c_{t}}=tanh\left(W_{c}^{T}x_{t}+U_{c}^{T}h_{t-1}+b_{c}\right)$$
- Forget Gate ($f_{t}$): sigmoid layer that takes into account the previous output and current input of the cell to determine which information from the cell memory has to be reset. Mathematically, it is described as follows:
  (4) $$f_{t}=\sigma \left(W_{f}^{T}x_{t}+U_{f}^{T}h_{t-1}+b_{f}\right)$$
- Output Gate ($o_{t}$): sigmoid layer that takes into account the previous output and current input of the cell to determine output candidate values. They are computed as:
  (5) $$o_{t}=\sigma \left(W_{o}^{T}x_{t}+U_{o}^{T}h_{t-1}+b_{o}\right)$$

Finally, the cell state and outputs are computed by accounting for the outputs of the above-mentioned gates and state candidates:

(6) $$c_{t}=f_{t}\circ c_{t-1}+i_{t}\circ \tilde{c_{t}}$$

(7) $$h_{t}=o_{t}\circ tanh\left(c_{t}\right)$$

Concerning the elements involved in the mathematical description of the LSTM cell, W, U, and b are the considered ANN’s weights and biases. The first (W) affects the cell’s input values and the second (U) affects the cell’s previous outputs. σ and tanh consist of the sigmoid and hyperbolic tangent activation functions. (∘) in Equations (6) and (7) corresponds to the Hadamard product. Finally, the last stacked LSTM cell output is adopted by a fully connected FFNN that generates a prediction by means of its weights, $W_{out}$, and biases, $b_{out}$. Equation (8) shows the soft sensor output value computation (i.e., the effluent prediction).

(8) $${\hat{y}}_{t}=f\left(W_{out}^{T}h_{t}+b_{out}\right)$$

### 2.5. Modeling

The modeling of the whole system was performed using different tools. Depending on their purpose, two different types of tools are defined: (i) the tools devoted to generating influent and effluent data; (ii) the tools considered in the ANN-based soft sensor’s training process.

The former consists mainly of the BSM2 framework, which was adopted to generate the influent, environmental, and effluent data. It was modified by adding the proposed MPC + fuzzy logic control structures proposed in [17]. Both the BSM2 framework and control strategies were emulated on the SIMULINK^®^ platform [18]. Notice that these data were used to generate the training, validation, and test sets for the ANN-based soft sensor’s training process.

On the other hand, data preprocessing and the considered ANNs were implemented using Python 2.7 language. Table 3 shows the different available and open-source libraries adopted in this work: Pandas [30], Scipy [31], and Numpy [32] were adopted to load and manage the data, and Matplotlib [33] was used to generate graphics. Scikit-Learn [34] was adopted in the K-Fold implementation, and TensorFlow [35] was used in the implementation of the ANN. In addition, LSTM’s training was carried out using NVIDIA^®^ GeForce RTX 2080 Titan GPU memory.

## 3. Effluent Concentrations and Alarm Prediction System

**Figure 7:**
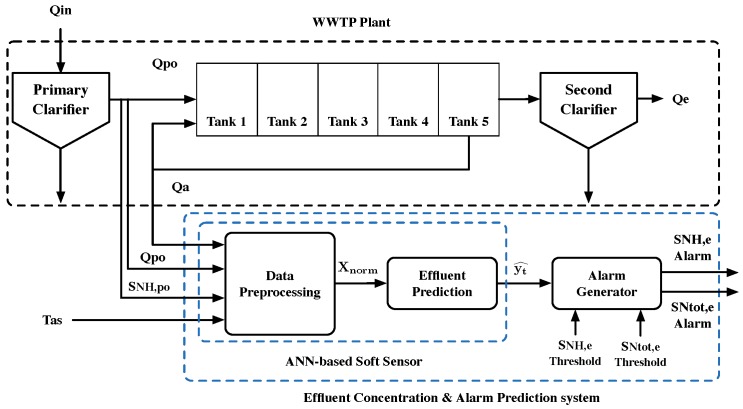
Effluent Concentrations and Alarm Prediction System (ECAPS) system. It generates an alarm whenever a violation of $S_{NH,e}$ or $S_{Ntot,e}$ limits is predicted.

Although there are several works in the literature that have implemented different control strategies to reduce WWTP effluent violations, violations still occur. Consequently, the overall cost of the WWTP not only increases from the application of control strategies but also as a result of the produced violations. In this work, we propose the Effluent Concentrations and Alarm Prediction System (ECAPS), whose goal is to track the effluent concentrations by means of an ANN-based soft sensor and generate alarms whenever a violation of the effluent limits is predicted. Among the different effluent concentrations, ECAPS focuses on the prediction of $S_{Ntot,e}$ and $S_{NH,e}$, two of the most difficult pollutants to reduce [17]. The former is a limiting nutrient and can therefore cause eutrophication, while the latter is toxic to aquatic life. ECAPS’ structure (see Figure 7) is based on two main blocks: the ANN-based soft sensor, which includes the Data Preprocessing and Effluent Prediction, and the Alarm Generation.

### 3.1. Data Preprocessing Block

**Figure 8:**
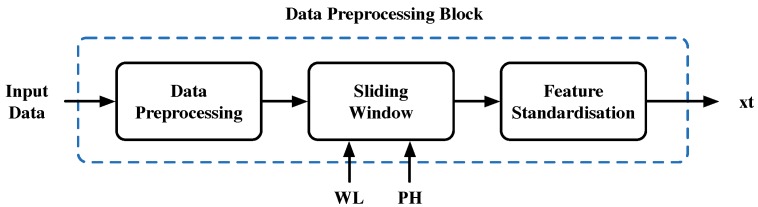
Data Preprocessing block. The three main actions performed in this block are shown.

The Data Preprocessing block (see Figure 8) is in charge of the data gathering and preprocessing process, which is briefly introduced in Section 2.3. Its main purpose is to gather the measurements of the different sensors distributed throughout the WWTP. Once they are gathered, the previously mentioned sliding window is applied. It is characterized by its window length (WL) and prediction horizon (PH), which are 10 and 4 h, respectively. Thus, the sum of WL and PH corresponds to the average WWTP retention time (14 h). Measurements are also normalized in the Data Preprocessing block. As a summary, the Data Preprocessing block prepares the data to feed the Effluent Prediction block.

### 3.2. Effluent Prediction Block

The Effluent Prediction is the block whose goal is to generate the predictions ($\hat{y_{t}}$) of the effluent nutrients’ concentrations. For the purpose of this work, those predictions correspond to the ammonium and total nitrogen concentrations, $S_{NH,e}$ and $S_{Ntot,e}$. This block consists of the proposed ANN-based soft sensor’s prediction part, where ANNs and especially LSTM cells are used in the predictive approach. These use WWTP measurements from the last 10 h (input vector generated at the Data Preprocessing Block) as inputs and generate a prediction of what the effluent concentrations will be in 4 h.

#### 3.2.1. Prediction Structures

Two prediction structures per control level were adopted: one to predict the ammonium concentration in the effluent ($S_{NH,e}$) and one for the total nitrogen concentration in the effluent ($S_{Ntot,e}$). Each prediction structure consists of two stacked-LSTM cells (see Figure 6) and an output network consists of a unique neuron, with one output adopting the linear activation function. The difference between structures is in the number of hidden neurons at each LSTM’s gate and the L2 penalty. The hyperparameter optimization process is performed to find the best LSTM structure in terms of L2 penalty and number of hidden neurons [36]. The process is based on training different configurations in which the applied hyperparameters (configuration variables external to the model and defined by the user) are varied in order to find those yielding the best performance. After performing the hyperparameter optimization process, a total of six prediction structures, two per control level, are obtained (see Table 4).

#### 3.2.2. Training Process

**Figure 9:**
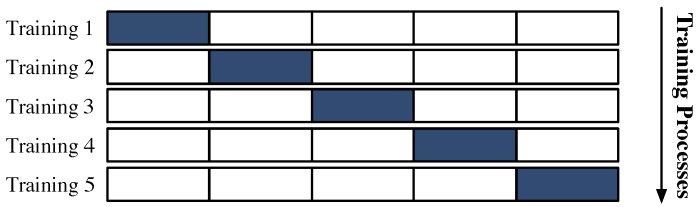
Application of K-Fold. Example of a K-Fold cross-validation; in each training process, the colored subset was adopted as the test data.

Prediction structures were trained with cross-validation, specifically the K-Fold technique [37]. This technique is based on implementing different data divisions in the ANN’s training process (the number of training runs is that same as the number of folds, so K experiments will be performed). In each experiment, the data subset devoted to testing the ANN performance is changed among the different equally sized subsets (K subsets) (see Figure 9) [37]. Consequently, K different model parameters per structure are obtained. For the purpose of this work, the K value was set to 5 just to ensure that at least 80% of the measurements were considered in the training dataset; the remaining measurements were used for test purposes. Half of these measurements generated the validation dataset. Validation data were used to assess overfitting.

Once training is finished, the structure’s performance can be obtained by either computing the average of the different experiments or choosing the model parameters that perform the best. Other approaches retrain a new model using the same settings of the best model parameters considered in the cross-validation [37,38]. In our case, the best model parameters were considered and consequently adopted in the prediction process. Moreover, the K-Fold technique was adopted in the training process not only to find the best model parameters but also to overcome the unbalanced dataset problem. It implicitly determines a good data division with which to train the network.

### 3.3. Alarm Generator Block

The Alarm Generator contrasts predictions with the effluent limits ($\hat{y_{t}}\geq \gamma$, where γ corresponds to the limits of either $S_{NH,e}$ ($\gamma_{NH,e}$) or $S_{Ntot,e}$ ($\gamma_{Ntot,e}$), see Table 1). Whenever those limits are violated (a violation is likely to be committed), an alarm is generated. Thus, the operator of the WWTP can decide to actuate against this future violation or not. In addition, the Alarm Generator can be calibrated taking into account the operators decision. For instance, if predictions are fully trusted, the Alarm Generator can be calibrated without modifying the limits. On the other hand, if predictions are not trusted, the Alarm Generator limits ($\gamma_{NH,e}$ and $\gamma_{Ntot,e}$) can be lowered, thus generating more alarms. However, an increase in false positives (predicting a violation when it does not occur) can result when the Alarm Generator limits are lowered. Calibration of the Alarm Generation is performed using receiver operating characteristic (RoC) curves, where the false positive rate is compared with the true positive rate or probability of detection (predicting a limit violation when it really occurs).

### 3.4. ECAPS Performance Evaluation

The performance of the proposed ECAPS was evaluated by means of five metrics: three related to the ANN-based soft sensor and two related to the alarm generation process. The ANN-based soft sensor’s performance was computed according to the mean absolute percentage error (MAPE), the root-mean-squared error (RMSE), and the determination coefficient ($R^{2}$). The Alarm Generator’s performance was computed on the basis of the false positive rate or false alarm probability ($P_{fa}$) and the real positive detection rate or probability of detection ($P_{d}$).

The MAPE is defined as the percentage of error in the prediction. Good predictions have MAPE values close to 0. It is computed as follows:

(9) $$\mathrm{MAPE}=\frac{1}{N}\cdot \sum_{i=1}^{N}\left|\frac{y_{i}-\hat{y_{i}}}{y_{i}}\right|\times 100$$

where N corresponds to the number of examples, $y_{i}$ corresponds to the *i*th sample of the real output data, and $\hat{y_{i}}$ is the *i*th predicted value. RMSE is computed as:

(10) $$\mathrm{RMSE}=\sqrt{\frac{1}{N}\sum_{i=1}^{N}{\left(y_{i}-\hat{y_{i}}\right)}^{2}}$$

Desirable RMSE values are close to 0.

The $R^{2}$ criterion is computed as:

(11) $$R^{2}=\frac{{\left(\sum_{i=1}^{N}\left(\hat{y_{i}}-\bar{\hat{y}}\right)\cdot \left(y_{i}-\bar{y}\right)\right)}^{2}}{\sum_{i=1}^{N}{\left(\hat{y_{i}}-\bar{\hat{y}}\right)}^{2}\cdot \sum_{i=1}^{N}{\left(y_{i}-\bar{y}\right)}^{2}}$$

where $\bar{\hat{y}}$ corresponds to the mean of the predicted values. The mean of the real values corresponds to $\bar{y}$. $R^{2}$ measures the amount of the data variance that is explained by the model and ranges from 0 to 1. Thus, a result of 1 reveals a perfect correlation between values.

Finally, the Alarm Generator’s purpose is based on the generation of alarms whenever the effluent concentrations exceed the limits. Thus, $P_{d}$ is defined as the probability of predicting existent limit violations. $P_{fa}$ is defined as the probability of predicting a nonexistent effluent limit violation. More specifically, $P_{fa}$ and $P_{d}$ are computed as:

(12) $$P_{fa}=P\left(\hat{y_{i}}\geq \gamma |y_{i}<\gamma \right)$$

(13) $$P_{d}=P\left(\hat{y_{i}}\geq \gamma |y_{i}\geq \gamma \right)$$

Predictions of nonexistent violations imply that actions are taken to reduce pollutant concentrations when they are not really required. Consequently, the overall cost of the WWTP is increased. For that reason, we aim for high $P_{d}$ and low $P_{fa}$ values.

## 4. Results

### 4.1. Hierarchical Control Prediction Structures Results

#### 4.1.1. ANN-Based Soft Sensor’s Prediction Results

**Figure 10:**
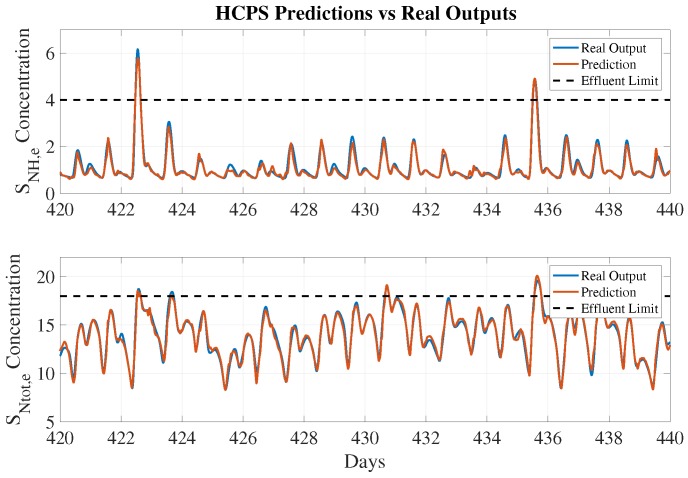
HCPS predictions vs. real outputs.

Results of the effluent prediction performance for the HCPS-NH and HCPS-NT are shown in Table 5. The five metrics previously presented determine which data split (fold) offers the best performance in terms of predictions, i.e., MAPE, RMSE, and $R^{2}$. Thus, the model parameters obtained in the corresponding fold will be adopted for the prediction process. In other words, each row in Table 5 shows the results obtained with all data by using the model parameters tuned at each fold. In our experiments (see Table 5), the second fold performs the best in terms of MAPE, RMSE, and $R^{2}$. However, it does not offer the best performance in terms of $P_{d}$ (around 87.63% for $S_{Ntot,e}$ and 86.57% for $S_{NH,e}$) and $P_{fa}$ (around 0.15 for $S_{Ntot,e}$ and 0.02 for $S_{NH,e}$). Nevertheless, they are very close to the highest $P_{d}$ and $P_{fa}$ values of around 87.63% and 0.15% for $S_{Ntot,e}$ and 91.05% and 0% for $S_{NH,e}$. Although predictions performed with a 2-fold data split are quite accurate (MAPE = 5.96% and RMSE = 0.12), some of them are below the real observed value. Consequently, there is a miss-detection, and thus, a violation of effluent limits occurs. Predictions performed by HCPS-NH and HCPS-NT are shown in Figure 10.

**Figure 11:**
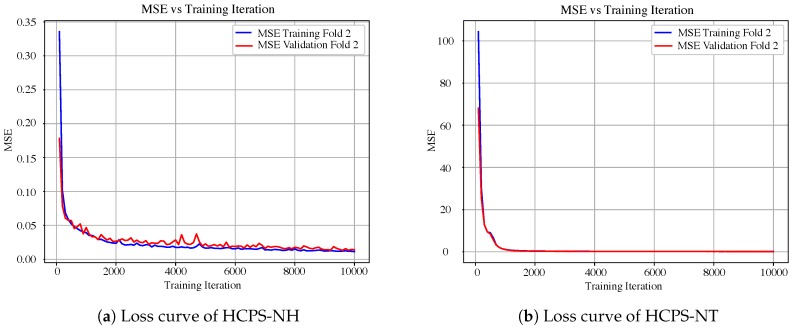
Loss curves for HCPS structures. Loss values correspond to MSE values from comparing real and predicted outputs throughout the training iterations.

In addition, the MSE error committed during the training process allows us to determine whether overfitting occurs. This is assessed by means of the validation and training curves, where the MSE value is computed at each training iteration (see Figure 11). Overfitting is deemed not to occur since an offset between curves is not observed. Moreover, both curves also show the speed of convergence. The less time the curve takes to reach a constant value, the quicker the convergence. For instance, it is observed that $S_{Ntot,e}$’s prediction model converges faster than $S_{NH,e}$’s.

It is worth noting that the implementation of a unique neural network to predict both concentrations ($S_{NH,e}$ and $S_{Ntot,e}$) was proposed and tested. However, the predictions did not yield good enough results in comparison with the performance of separated networks (dedicated networks). For instance, the best model presents an RMSE of around 0.62 and 0.15 in terms of $S_{Ntot,e}$ and $S_{NH,e}$ predictions. If the results are contrasted in terms of the determination coefficient, the difference between the performances of the prediction structures is even larger. In terms of $S_{NH,e}$ predictions, a unique network shows a determination coefficient of around 0.88, whilst the separated structure, HCPS-NH, shows a determination coefficient of around 0.93. For that reason, this work used two separated ANN, one for each case.

#### 4.1.2. Alarm Generator Performance

Predictions are fed into the Alarm Generator to determine whether a future violation will occur. As observed in Table 5, the probabilities of detection $P_{d}$ and false alarm $P_{fa}$ were computed according to BSM2 limits (violations are produced if $S_{NH,e}\geq$ 4 mg/L and $S_{Ntot,e}\geq$ = 18 mg/L). Thus, one can observe that leaving the thresholds as such implies that the probabilities of detection are still low for the purpose of hazard prevention. For that reason, the Alarm Generator was provided with two degrees of freedom to allow the WWTP’s operator to adjust the violation detection sensitivity. Those degrees of freedom correspond to the thresholds adopted by the Alarm Generator ($\gamma_{NH,e}$ and $\gamma_{Ntot,e}$). In this context, a decrease in the ECAPS’ thresholds is translated into an improvement in the probability of detection, making the system able to detect more effluent violations. However, this lowering also implies an increase in false positives. This is translated into an increase in the WWTP’s operational cost because actions are taken to reduce the pollutant concentrations when they are not necessary.

**Figure 12:**
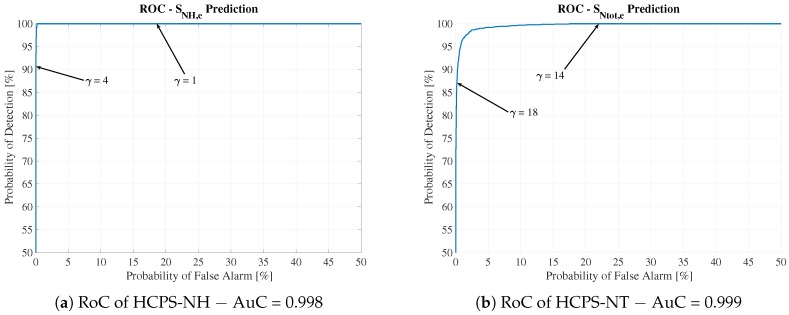
Receiver operating characteristic (RoC) curves for HCPS structures.

Thus, the Alarm Generator sensitivity can be calibrated by the WWTP’s operator following the well-known RoC. RoC curves are obtained by plotting $P_{fa}$ and $P_{d}$. Therefore, these curves can be understood as a tool that relates the probability of detection to the false positive rate. They are computed by varying the ECAPS’ thresholds ($\gamma_{NH,e}$ and $\gamma_{Ntot,e}$) and taking the new detection and false alarm probabilities. In addition, the area under the curve (AuC) is computed as a metric that indicates the Alarm Generator’s performance. The closer to 1 the AuC, the better the performance. RoC curves for HCPS are shown in Figure 12, where one can observe the behavior of the Alarm Generator when its thresholds, γ, are varied. Figure 12a shows that a variation in the effluent threshold from 4 to 1 implies an increase not only in the probability of detection but also in the false positive rate. In other words, the rates are 86.57% for detection and 0.02% for false positive for $\gamma_{NH,e}$ = 4. For $\gamma_{NH,e}$ = 1, $P_{d}$ and $P_{f}$ are 100% and 18.38%, respectively. From the WWTP’s operational point of view, all future violations will be detected and therefore addressed by applying the correspondent control strategies. However, the WWTP’s overall cost will be increased by false positive violations. Thus, the operator of the plant has to deal with this trade-off: decide to detect all possible violations, even though false positives are also more likely to occur (the operational WWTP cost is increased), or assume some possible violations without increasing the operational cost.

### 4.2. Default Control and Open Loop Prediction Structures Results

Since ECAPS is a system that can be applied to WWTPs for different control strategies, it was also tested with DC (Default Control) [4] and OL (Open Loop) control strategies. In such a context, prediction structures have to be retrained in order to get models that fit DC and OL data. Those structures correspond to DCPS and OLPS.

#### 4.2.1. ANN-Based Soft Sensor’s Prediction Results

The DCPS and OLPS performance is shown in Table 6, where the performances of the different training runs are shown. As was performed with HCPS structures, the fold (data split) that offers the best performance is the one determining the model parameters adopted for predicting purposes. A fold of 2 was appropriate for the DCPS-NH, OLPS-NH, and OLPS-NT structures, whereas a fold of 1 was best for DCPS-NT.

**Figure 13:**
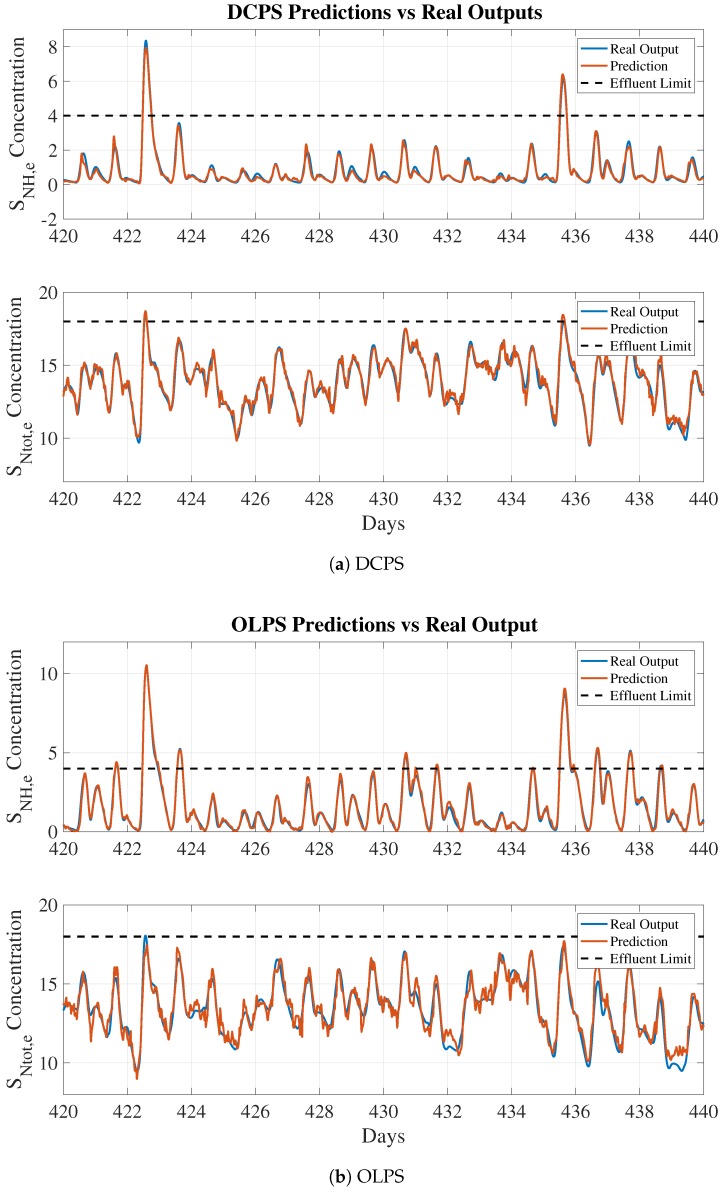
DCPS and OLPS performed predictions vs. real outputs.

Concerning the results of structures predicting $S_{NH,e}$, the performance of DCPS-NH and OLPS-NH is worse compared with that of HCPS-NH. For instance, the MAPE of HCPS-NH is 5.96%, whereas that of OLPS-NH is 21.62%. This leads to the notion that the structures are generating very poor predictions. However, if the MAPE is complemented with the RMSE, one can observe that the performance degradation is not so severe. The RMSEs of DCPS-NH and OLPS-NH are equal to 0.15 and 0.28, respectively, whereas HCPS-NH’s RMSE equals 0.12. In addition, the probabilities of detecting violations are improved: the lowest probability is now offered by DCPS-NH (89.02%), whereas HCPS-NH’s probability of violation detection equals 86.57% when maintaining the thresholds at the BSM2 limits. In terms of the structures predicting $S_{Ntot,e}$, significant performance degradation is observed. Although RMSE values for the DCPS-NT and OLPS-NT structures are lower than the HCPS-NT RMSE, the MAPE and $R^{2}$ values are higher, meaning that the predictions have been made worse. The difference is even larger when observing probabilities of violation detection. They equal 68.10% and 64.22% for DCPS-NT and OLPS-NT structures, respectively, whereas it equals 85.96% for HCPS-NT. In this case, the probability of violation detection has been degraded by between 20.77% and 25.29%. However, if the predictions are observed (see Figure 13), one can see that the predicted values are not so different from the target values. Thus, the proposed soft sensor can be adopted to predict effluent concentrations in different control scenarios at the expense of improving the violation detection by calibrating ECAPS’ Alarm Generator block.

From an operational point of view, performance degradation is highly related to the control strategies adopted in the different scenarios (HC, DC, and OL). They have a direct impact on whether the variability in effluent concentration decreases or increases: the biggest ranges and peaks of $S_{NH,e}$ are observed in the OL scenario, whereas the biggest ones for $S_{Ntot,e}$ are observed in the HC scenario. This is a result of the reduction in ammonium in the nitrification process, which increases the nitrates and therefore the total nitrogen amount. Consequently, a less sophisticated control mechanism (DC) or no control (OL) induces more variability in effluent signals. For this reason, prediction becomes more problematic and ANN performs worse.

In addition, the MSE from the training process allows us to determine whether overfitting is occurring. This is performed by means of the validation and training curves, where the MSE value is computed for each training iteration. As a result, two curves are obtained, as seen in Figure 11, where 2-fold validation and training curves are shown. As is observed, overfitting is not an issue since an offset between curves is not observed. Moreover, both curves also show the speed of convergence. The less time the curve takes to reach a constant value, the quicker the convergence. For·instance, it is observed that the $S_{Ntot,e}$ prediction model converges faster than the $S_{NH,e}$ model.

**Figure 14:**
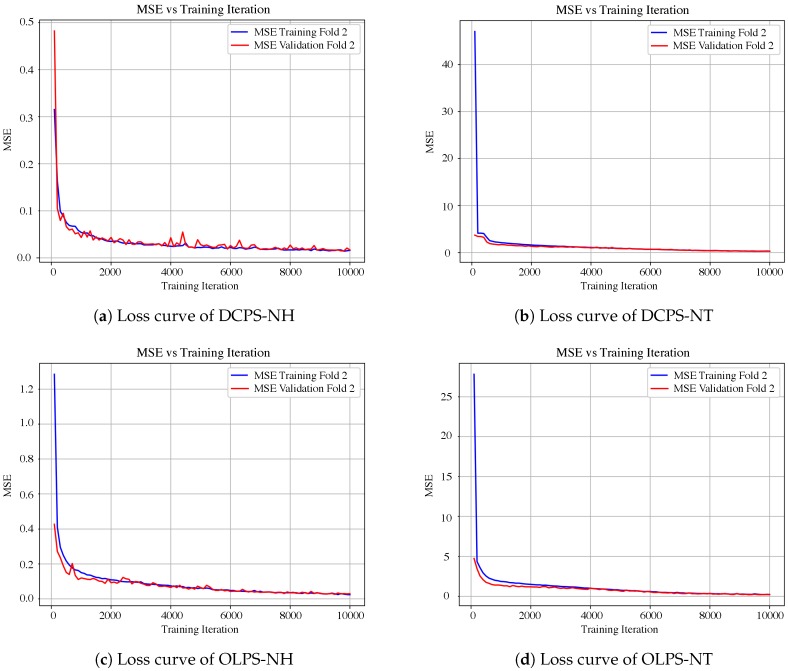
Loss curves for DCPS and OLPS structures. Loss values correspond to MSE values between real and predicted outputs throughout the training iterations.

Finally, Figure 14 shows the MSE for each training iteration for DCPS-NH, DCPS-NT, OLPS-NH, and OLPS-NT. As is observed, no overfitting occurs. Therefore, the model parameters tuned at the corresponding fold can be used to form predictions. Moreover, one can observe that the structures predicting $S_{Ntot,e}$ take more time to converge since the MSE at the very beginning is higher than the MSE of the structures predicting $S_{NH,e}$.

#### 4.2.2. Alarm Generator Performance

As has been observed previously, the structures predicting $S_{NH,e}$ for the DC and OL scenarios have a performance similar to that of HCPS-NH. The probabilities of violation equal 89.02% and 86.57% for DCPS-NH and OLPS-NH, respectively. They are even greater than HCPS-NH. However, the contrary occurs when dealing with structures predicting $S_{Ntot,e}$, where the probabilities of violation detection are highly degraded. Therefore, these probabilities have to be improved if they are going to be used to predict effluent concentrations in the DC and OL scenarios. Their improvement is performed by varying the Alarm Generator’s thresholds ($\gamma_{NH,e}$ and $\gamma_{Ntot,e}$).

**Figure 15:**
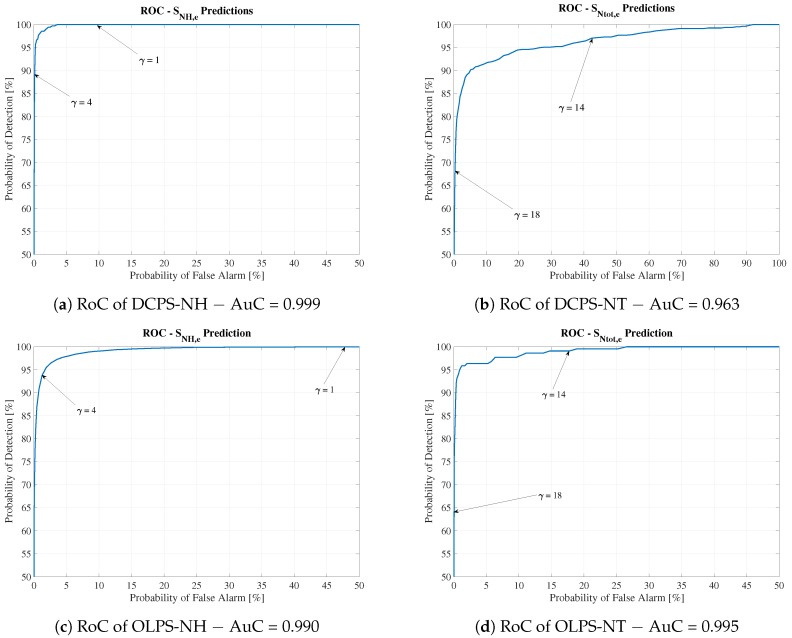
RoC curves for DCPS and OLPS structures.

The RoC curves showing the values of detection and false alarm probabilities were also computed. They show that although the probability of detection worsens in some cases (the highest degradation of detection probability corresponds to 25.29%), the soft sensor can still be adopted because the RoC curves show high AuC values (see Figure 15). For instance, the OLPS-NH RoC curve yields an AuC of around 0.990, and the OLPS-NT RoC curve has an AuC of around 0.995. This is translated into the fact that the probability of detecting $S_{NH,e}$ violations can be improved to 100% detection by lowering the Alarm Generator $\gamma_{NH,e}$ threshold from 4 to 1. However, the false positive rate is also increased: the false alarm probability is increased from 1.25% to 47.64%. The same principle applies to the probability of detecting $S_{Ntot,e}$ violations. Changing the effluent threshold from 18 to 14 is translated into improving the probability of detection from 64.22% to 99.08%. The false positive rate is increased from 0.04% to 17.74%. Regarding the DCPS scenario, the DCPS-NH RoC curves show an AuC of 0.999, whilst the DCPS-NT curve corresponds to an AuC of 0.963. In both cases, a decrease in the effluent thresholds improves the probability of detection but also increases the false positive rates.

Finally, it is worth noting that ECAPS was deployed with BSM2 WWTPs to detect future violations of effluent concentrations and also to feed control strategies that will actuate over biological reactors to reduce the pollutant concentrations. However, the BSM2 WWTP structure considers the implementation of bypass events, which are events in which water is directly spilled into the WWTP’s second clarifier without going through the biological reactors. Thus, violations can be performed since this water has not been treated in the biological reactors. These events are produced when influent suddenly increases and WWTP detects that an overflow will be produced [4]. Consequently, ECAPS will have not predicted these violations because the influent measurements it adopts come from the primary clarifier, whilst bypass is performed before the primary clarifier. For that reason, some violations can still occur. They can be reduced by implementing new control strategies responsible for keeping secondary clarifier concentrations under the considered limits. However, this is out of the scope of this work.

## 5. Conclusions

This work is based on the implementation of an Effluent Concentration and Alarm Prediction System (ECAPS) whose aim is to predict concentrations of a WWTP’s effluent and determine when they will exceed the established limits. Since it will be deployed in the BSM2 framework, the limits correspond to BSM2’s own regulations. Predictions are performed by means of an ANN-based soft sensor system. Among the different effluent concentrations, ECAPS is focused on predicting $S_{NH,e}$ and $S_{Ntot,e}$ concentrations. An Alarm Generator system compares the predictions with real imposed effluent limits to determine when future violations of the WWTP’s limits will occur. Consequently, the WWTP operator will be able to act in advance to prevent the predicted violation.

ECAPS predictions are performed by means of two LSTM-based structures, one per effluent considered. Moreover, the ANN-based soft sensor system was designed for three different levels of control, i.e., high, medium, and low control. These levels, as well as the BSM2 framework, were used in the data generation process. The training process was performed by means of the K-fold technique in order to mitigate the problem of highly unbalanced datasets.

Results show that high-accuracy predictions can be obtained for those structures predicting ammonium ($S_{NH,e}$) concentrations. The probability of violation detection is around 86.57%, 89.02%, and 93.77% for high, medium, and low control levels. In terms of structures devoted to predicting $S_{Ntot,e}$ concentration, their performance shows that good levels of violation detection are achieved when a high control level is used (around 85.96%). Moreover, the probabilities of violation detection can be improved by decreasing the ECAPS effluent thresholds at the expense of increasing the false positive rates. RoC curves were computed as a means to select these thresholds. They show an AuC of around 0.99, which translates to a nearly perfect performance. In that vein, 100% of the violations can be detected.

## Figures and Tables

**Table 1:** BSM2 effluent quality limits.

Effluent Quality Limits	
Variable	Value
$S_{Ntot,e}$	<18 mg/L
COD	<100 mg/L
$S_{NH,e}$	<4 mg/L
TSS	<30 mg/L
$BOD_{5}$	<10 mg/L

**Table 2:** Range, mean, and standard deviation of some WWTP measurements.

**Input Measurements**	
**Measurement**	**Description**
**Influent and Internal measurements**	
$S_{NH,po}$ (mg/L)	Primary clarifier’s ammonium concentration
$Q_{po}$ (m${}^{3}$/day)	Primary clarifier’s overflow rate
$Q_{a}$ (m${}^{3}$/day)	Internal recycle flow
**Environmental measurements**	
$T_{as}$ (°C)	Environment Temperature
**Output measurements**	
**Effluent measurements**	
$S_{Ntot,e}$ (mg/L)	Effluent’s total nitrogen concentration
$S_{NH,e}$ (mg/L)	Effluent’s ammonium concentration

**Table 3:** Python 2.7’s libraries used in this work.

Python 2.7 Libraries		
Library	Purpose	Version
Pandas [30]	Data Loading	0.23.4
Scipy [31]	Data Loading	1.1.0
Numpy [32]	Data Management	1.15.4
Matplotlib [33]	Graphic Generation	2.2.2
Scikit-learn [34]	K-Fold implementation	0.20.1
TensorFlow [35]	ANNs net implementation	1.12.0

**Table 4:** Obtained prediction structures (PS). Information related to the number of hidden neurons, L2 penalty, and level of control can be observed.

**LSTM Structures**			
**Hierarchical Control Level**			
Structure	Output concentration	Hidden Neurons per Gate	L2 Penalty
HCPS-NH	$S_{NH,e}$	50	$1\times 10^{-3}$
HCPS-NT	$S_{Ntot,e}$	40	$1\times 10^{-3}$
**Default Control Level**			
Structure	Output concentration	Hidden Neurons per Gate	L2 Penalty
DCPS-NH	$S_{NH,e}$	75	$1\times 10^{-3}$
DCPS-NT	$S_{Ntot,e}$	100	$1\times 10^{-3}$
**Open Loop Level**			
Structure	Output concentration	Hidden Neurons per Gate	L2 Penalty
OLPS-NH	$S_{NH,e}$	100	$1\times 10^{-3}$
OLPS-NT	$S_{Ntot,e}$	125	$5\times 10^{-3}$

**Table 5:** Performance of HCPS structures adopting the K-Fold technique. Results in bold correspond to the fold offering the best performance in terms of the mean absolute percentage error (MAPE) and root-mean-square error (RMSE).

**HCPS Performance**					
**HCPS-NH Performance**					
**Fold**	**MAPE (%)**	**RMSE**	$R^{2}$	**$P_{\mathrm{fa}}$ (%)**	**$P_{d}$ (%)**
1	6.77	0.18	0.83	0.11	63.43
**2**	**5.96**	**0.12**	**0.93**	**0.02**	**86.57**
3	6.71	0.12	0.93	0.02	91.05
4	6.45	0.15	0.89	0.00	70.90
5	6.04	0.14	0.90	0.01	70.90
**HCPS-NT Performance**					
**Fold**	**MAPE (%)**	**RMSE**	$R^{2}$	**$P_{\mathrm{fa}}$ (%)**	**$P_{d}$ (%)**
1	2.57	0.44	0.98	0.35	87.50
**2**	**2.43**	**0.40**	**0.98**	**0.15**	**85.96**
3	2.71	0.44	0.98	0.20	87.63
4	2.71	0.44	0.98	0.30	86.03
5	2.65	0.44	0.98	0.25	83.36

**Table 6:** Performance of DCPS and OLPS structures after applying the K-Fold technique. Results in bold correspond to the fold that offers the best performance in terms of the MAPE and RMSE.

**DCPS Performance**					
**DCPS-NH Performance**					
**Fold**	**MAPE (%)**	**RMSE**	$R^{2}$	**$P_{\mathrm{fa}}$ (%)**	**$P_{d}$ (%)**
1	23.23	0.25	0.87	0.27	75.43
**2**	**22.65**	**0.15**	**0.95**	**0.10**	**89.02**
3	23.82	0.15	0.95	0.02	88.44
4	23.26	0.19	0.92	0.10	73.12
5	23.01	0.18	0.93	0.09	84.10
**DCPS-NT Performance**					
**Fold**	**MAPE (%)**	**RMSE**	$R^{2}$	**$P_{\mathrm{fa}}$ (%)**	**$P_{d}$ (%)**
**1**	**3.74**	**0.84**	**0.84**	**0.36**	**68.10**
2	4.12	0.86	0.83	0.46	65.28
3	4.40	0.91	0.81	0.27	57.67
4	4.31	0.88	0.83	0.41	69.57
5	4.57	0.90	0.95	0.10	53.37
**OLPS Performance**					
**OLPS-NH Performance**					
**Fold**	**MAPE (%)**	**RMSE**	$R^{2}$	**$P_{\mathrm{fa}}$ (%)**	**$P_{d}$ (%)**
1	23.63	0.33	0.96	0.98	88.02
**2**	**21.62**	**0.28**	**0.97**	**1.25**	**93.77**
3	25.51	0.29	0.96	0.97	94.46
4	23.22	0.28	0.97	1.47	94.07
5	22.74	0.33	0.96	0.73	90.25
**OLPS-NT Performance**					
**Fold**	**MAPE (%)**	**RMSE**	$R^{2}$	**$P_{\mathrm{fa}}$ (%)**	**$P_{d}$ (%)**
1	4.81	0.81	0.86	0.03	30.28
**2**	**4.41**	**0.75**	**0.88**	**0.04**	**64.22**
3	4.99	1.04	0.77	0.04	47.71
4	4.66	0.82	0.86	0.02	57.80
5	4.56	0.77	0.87	0.02	63.20

## Abbreviations

The following abbreviations and symbols are used in this work:

ANN	Artificial Neural Network
ASM1	Activated Sludge Model N.1
ASM2	Activated Sludge Model N.2
ASM2d	Activated Sludge Model N.2d
AuC	Area under the Curve
$BOD_{5}$	Biochemical Oxygen Demand (mg/L)
BP	Back-propagation
BPTT	Back-propagation Through Time
BSM1	Benchmark Simulation Model N.1
BSM2	Benchmark Simulation Model N.2
COD	Chemical Oxygen Demand
DC	Default Control Scenario
DCPS	Default Control Prediction Structure
DCPS-NH	DCPS for $S_{NH,e}$
DCPS-NT	DCPS for $S_{Ntot,e}$
ECAPS	Effluent Concentration & Alarm Prediction System
FFNN	Feed-forward Neural Network
FG	LSTM’s Forget Gate
HC	Hierarchical Control scenario
HCNH	Hierarchical Control for Ammonium
HCNtot	Hierarchical Control for Total Nitrogen
HCPS	Hierarchical Control Prediction Structure
HCPS-NH	HCPS for $S_{NH,e}$
HCPS-NT	HCPS for $S_{Ntot,e}$
HP	Horizon of prediction
IG	LSTM’s Input Gate
IoT	Internet of Things
IWA	International Water Association
$K_{l}a$*x*	Oxygen transfer coefficient for tank *x* (day${}^{-1}$)
LSTM	Long-Short Term Memory
MAPE	Mean average percentage error
MLP	Multilayer perceptron
MPC	Model predictive control
MSE	Mean-squared error
OG	LSTM’s Output Gate
OL	Open Loop scenario
OLPS	Open Loop Prediction Structure
OLPS-NH	OLPS for $S_{NH,e}$
OLPS-NT	OLPS for $S_{Ntot,e}$
$P_{d}$	Detection probability
$P_{fa}$	False alarm probability
PI	Proportional integral controller
$q_{EC,x}$	External carbon flow rate at tank *x* (m${}^{3}$/day)
$Q_{a}$	Internal recirculation flow rate (m${}^{3}$/day)
$Q_{e}$	Effluent flow rate (m${}^{3}$/day)
$Q_{in}$	Influent flow rate (m${}^{3}$/day)
$Q_{po}$	Primary clarifier’s flow rate (m${}^{3}$/day)
$Q_{r}$	Sludge internal recycle flow rate (m${}^{3}$/day)
*R*	Correlation coefficient
$R^{2}$	Determination coefficient
RMSE	Root-mean-squared error
RNN	Recurrent neural network
ROC	Receiver operating characteristic
$S_{NH}$	Ammonium concentration (mg/L)
$S_{NH,e}$	Ammonium concentration in the effluent (mg/L)
$S_{Ntot}$	Total nitrogen concentration (mg/L)
$S_{Ntot,e}$	Total Nitrogen Concentration in the effluent (mg/L)
$S_{O,x}$	Dissolved oxygen concentrations at tank x (mg/L)
$S_{P,e}$	Total phosphorus concentration in the effluent (mg/L)
SS	Suspended solids (mg/L)
$T_{as}$	Environment temperature (°C)
TKN	Total Kjeldahl nitrogen (mg/L)
TSS	Total suspended solids (mg/L)
WL	Window length
WWTP	Wastewater treatment plant
${\hat{y}}_{t}$	ANN-based soft sensor’s predictions
γ	Alarm Generator’s threshold (mg/L)
$\gamma_{NH,e}$	$S_{NH,e}$ threshold (mg/L)
$\gamma_{Ntot,e}$	$S_{Ntot,e}$ threshold (mg/L)

## References

[1] European Commission (1991). Council Directive 91/271/EEC of 21 May 1991 concerning urban waste water treatment. J. Eur. Communities.

[2] Henze M., Grady L., Gujer W., Marais G.V.R., Matsuo T. (1987). Activated Sludge Model No 1. IAWPRC Sci. Tech. Rep..

[3] Copp J.B. (2002). The Cost Simulation Benchmark: Description and Simulator Manual (Cost Action 624 and Action 682).

[4] Gernaey K.V., Jeppsson U., Vanrolleghem P.A., Copp J.B., International Water Association, Task Group on Benchmarking of Control Strategies for Wastewater Treatment Plants (2014). Benchmarking of Control Strategies for Wastewater Treatment Plants.

[5] Vrecko D., Volcke E., Jeppsson U., Gernaey K. (2007). Evaluation Criteria Description and Example for BSM2. International Water Association; Task Group on Benchmarking of Control Strategies for Wastewater Treatment Plants.

[6] Goodfellow I., Bengio Y., Courville A. (2016). Deep Learning.

[7] Ou H.S., Wei C.H., Wu H.Z., Mo C.H., He B.Y. (2015). Sequential dynamic artificial neural network modeling of a full-scale coking wastewater treatment plant with fluidized bed reactors. Environ. Sci. Pollut. Res..

[8] Shen W., Chen X., Corriou J.P. (2008). Application of model predictive control to the BSM1 benchmark of wastewater treatment process. Comput. Chem Eng..

[9] Fortuna L., Graziani S., Xibilia M.G. (2005). Soft sensors for product quality monitoring in debutanizer distillation columns. Control Eng. Pract..

[10] Rani A., Singh V., Gupta J.R. (2013). Development of soft sensor for neural network based control of distillation column. ISA Trans..

[11] Fernandez de Canete J., Del Saz-Orozco P., Baratti R., Mulas M., Ruano A., Garcia-Cerezo A. (2016). Soft-sensing estimation of plant effluent concentrations in a biological wastewater treatment plant using an optimal neural network. Expert Syst. Appl..

[12] Wollschlaeger M., Sauter T., Jasperneite J. (2017). The future of industrial communication: Automation networks in the era of the internet of things and industry 4.0. IEEE Ind. Electron. Mag..

[13] Güçlü D., Dursun Ş. (2010). Artificial neural network modelling of a large-scale wastewater treatment plant operation. Bioprocess Biosyst. Eng..

[14] Nasr M.S., Moustafa M.A.E., Seif H.A.E., El Kobrosy G. (2012). Application of artificial neural network (ANN) for the prediction of EL-AGAMY wastewater treatment plant performance-EGYPT. Alex. Eng. J..

[15] Foscoliano C., Del Vigo S., Mulas M., Tronci S. (2016). Predictive control of an activated sludge process for long term operation. Chem. Eng. J..

[16] Manu D.S., Thalla A.K. (2017). Artificial intelligence models for predicting the performance of biological wastewater treatment plant in the removal of Kjeldahl Nitrogen from wastewater. Appl. Water Sci..

[17] Santín I., Pedret C., Vilanova R., Meneses M. (2016). Advanced decision control system for effluent violations removal in wastewater treatment plants. Control Eng. Pract..

[18] Jeppsson U., Pons M.N., Nopens I., Alex J., Copp J., Gernaey K., Rosen C., Steyer J.P., Vanrolleghem P. (2007). Benchmark simulation model no 2: General protocol and exploratory case studies. Water Sci. Technol..

[19] Yang P.Y., Zhang Z. Nitrification and denitrification in the wastewater treatment system. Proceedings of the UNESCO–University of Tsukuba International Seminar on Traditional Technology for Environmental Conservation and Sustainable Development in the Asian-Pacific Region.

[20] Takács I., Patry G.G., Nolasco D. (1991). A dynamic model of the clarification-thickening process. Water Res..

[21] Vilanova R., Santín I., Pedret C. (2017). Control y Operación de Estaciones Depuradoras de Aguas Residuales: Modelado y Simulación. RIAI Rev. Iberoam. Autom. Inform. Ind..

[22] Lorenzo-Toja Y., Vázquez-Rowe I., Amores M.J., Termes-Rifé M., Marín-Navarro D., Moreira M.T., Feijoo G. (2016). Benchmarking wastewater treatment plants under an eco-efficiency perspective. Sci. Total Environ..

[23] Henze M., Gujer W., Mino T., van Loosdrecht M.C. (2000). Activated Sludge Models ASM1, ASM2, ASM2d and ASM3.

[24] Vilanova R., Visioli A. (2012). PID Control in the Third Millennium—Lessons Learned and News Approaches.

[25] Grus J. (2015). Data Science from Scratch: First Principles with Python.

[26] Da Silva I.N., Spatti D.H., Flauzino R.A., Liboni L.H.B., dos Reis Alves S.F. (2017). Artificial Neural Networks.

[27] Gardner M., Dorling S. (1998). Artificial neural networks (the multilayer perceptron)—A review of applications in the atmospheric sciences. Atmos. Environ..

[28] Bengio Y., Simard P., Frasconi P. (1994). Learning Long-Term Dependencies with Gradient Descent is Difficult. IEEE Trans. Neural Netw..

[29] Greff K., Srivastava R.K., Koutnik J., Steunebrink B.R., Schmidhuber J. (2017). LSTM: A Search Space Odyssey. IEEE Trans. Neural Netw. Learn. Syst..

[30] McKinney W. Data structures for statistical computing in python. Proceedings of the 9th Python in Science Conference.

[31] Jones E., Oliphant T., Peterson P. (2001). SciPy: Open Source Scientific Tools for Python. http://www.scipy.org/.

[32] Oliphant T.E. (2006). A Guide to NumPy.

[33] Hunter J.D. (2007). Matplotlib: A 2D graphics environment. Comput. Sci. Eng..

[34] Pedregosa F., Varoquaux G., Gramfort A., Michel V., Thirion B., Grisel O., Blondel M., Prettenhofer P., Weiss R., Dubourg V. (2011). Scikit-learn: Machine learning in Python. J. Mach. Learn. Res..

[35] Abadi M., Agarwal A., Barham P., Brevdo E., Chen Z., Citro C., Corrado G.S., Davis A., Dean J., Devin M. (2015). TensorFlow: Large-Scale Machine Learning on Heterogeneous Systems. https://www.tensorflow.org/.

[36] Bengio Y. (2012). Practical Recommendations for Gradient-Based Training of Deep Architectures. Neural Networks: Tricks of the Trade.

[37] Bergmeir C., Hyndman R.J., Koo B. (2018). A note on the validity of cross-validation for evaluating autoregressive time series prediction. Comput. Stat. Data Anal..

[38] Rohani A., Taki M., Abdollahpour M. (2018). A novel soft computing model (Gaussian process regression with K-fold cross validation) for daily and monthly solar radiation forecasting (Part: I). Renew. Energy.

